# Emergence and evolution of inter-specific segregating retrocopies in cynomolgus monkey (*Macaca fascicularis*) and rhesus macaque (*Macaca mulatta*)

**DOI:** 10.1038/srep32598

**Published:** 2016-09-07

**Authors:** Xu Zhang, Qu Zhang, Bing Su

**Affiliations:** 1 State Key Laboratory of Genetic Resources and Evolution, Kunming Institute of Zoology, Chinese Academy of Sciences, Kunming 650223, China; 2 Kunming College of Life Science, University of Chinese Academy of Sciences, Beijing 100049, China; 3 Department of Human Evolutionary Biology, Graduate School of Art and Science, Harvard University, Cambridge, MA 02138, USA; 4 Perspective Sciences, 18 Qingfeng North Road, Phoenix Building A, North New Zone, Chongqing 401320, China

## Abstract

Retroposition is an RNA-mediated mechanism to generate gene duplication, and is believed to play an important role in genome evolution and phenotypic adaptation in various species including primates. Previous studies suggested an elevated rate of recent retroposition in the rhesus macaque genome. To better understand the impact of retroposition on macaque species which have undergone an adaptive radiation approximately 3–6 million years ago, we developed a bioinformatics pipeline to identify recently derived retrocopies in cynomolgus monkeys. As a result, we identified seven experimentally validated young retrocopies, all of which are polymorphic in cynomolgus monkeys. Unexpectedly, five of them are also present in rhesus monkeys and are still segregating. Molecular evolutionary analysis indicates that the observed inter-specific polymorphism is attribute to ancestral polymorphism. Further population genetics analysis provided strong evidence of balancing selection on at least one case (Crab-eating monkey retrocopy 6, or CER6) in both species. CER6 is in adjacent with an immunoglobulin related gene and may be involved in host-pathogen interaction, a well-known target of balancing selection. Altogether, our data support that retroposition is an important force to shape genome evolution and species adaptation.

Retroposition is a gene duplication mechanism mediated by an RNA intermediate, where new intronless retrocopies are derived by reverse transcription of messenger RNA (mRNA) and then integrated into random genomic regions^1^,^2^,^3^. In primates, L1 retrotransposons are believed to be the major player in this process, facilitated by reverse-transcriptase and endonuclease encoded by L1 ORF2 protein^4^,^5^,^6^. Retroposition events have been well characterized in the genome of primates and other species^7^,^8^,^9^,^10^,^11^, implying their potential role in shaping genome evolution.

Retrocopies originated by retroposition are generally considered as “dead on arrival” since they usually lack promoter sequences from their parental genes and are consequently inactive in transcription. However, a growing number of studies provided evidence that some retrocopies can become functional genes (retrogenes) and are subject to various evolutionary constraints^8^,^9^,^10^,^12^,^13^, or even associate with phenotypic traits in mammals^14^,^15^,^16^. Additionally, non-protein-coding retrocopies (retropseudogenes or processed pseudogenes) could also be expressed^17^ and become functional through expressing antisense transcripts to couple the mRNA of their parental genes^18^,^19^,^20^ or to buffer miRNAs^21^. Collectively, retrocopies are actively involved in various aspects of evolution.

As the most widespread non-human primate, the genus *Macaca* diversified approximately 5 million years ago^22^ and contains about 20 species spanning from northeast Africa to Asia^23^,^24^,^25^. Split from ape and human ancestors about 25 million years ago^26^, macaques are valuable animal models to understand human biology and are widely used in biomedical research. Our previous study in rhesus macaque (RM, *Macaca mulatta*) has identified a burst of retroposition events after its separation from great apes^10^. Detailed characterization of recent retropositions in the macaque genus may help us further understand their speciation and adaptive evolution, with potential significance in human evolutionary studies.

For a long time, the majority of published work on retroposition focus on species with well annotated genomes^8^,^9^,^10^,^13^. As a result of the fast development of next-generation sequencing technology, a substantial number of new genomes become available, providing an opportunity to study the pattern of retroposition in a broad scope. In this study, we exploited the deep sequencing data of cynomolgus or crab-eating macaque (CE, *Macaca fascicularis*), a model non-human primate animal commonly used in medical research and diverged from rhesus macaque about 3–6 million years ago^25^,^27^,^28^. We developed a bioinformatics framework to identify recent retrocopies in CE and studied their evolutionary pattern. In addition to polymorphic retrocopies within species, which have drawn substantial attentions recently^29^,^30^,^31^,^32^,^33^,^34^, we were surprised to discover inter-specific polymorphic retrocopies shared in two macaque species. Detailed evolutionary analyses suggest ancestral polymorphisms are the main origin of these inter-specific segregating retrocopies, and balancing selection may be one of the major forces maintaining them.

## Results

### Bioinformatics pipeline

**Figure 1:**
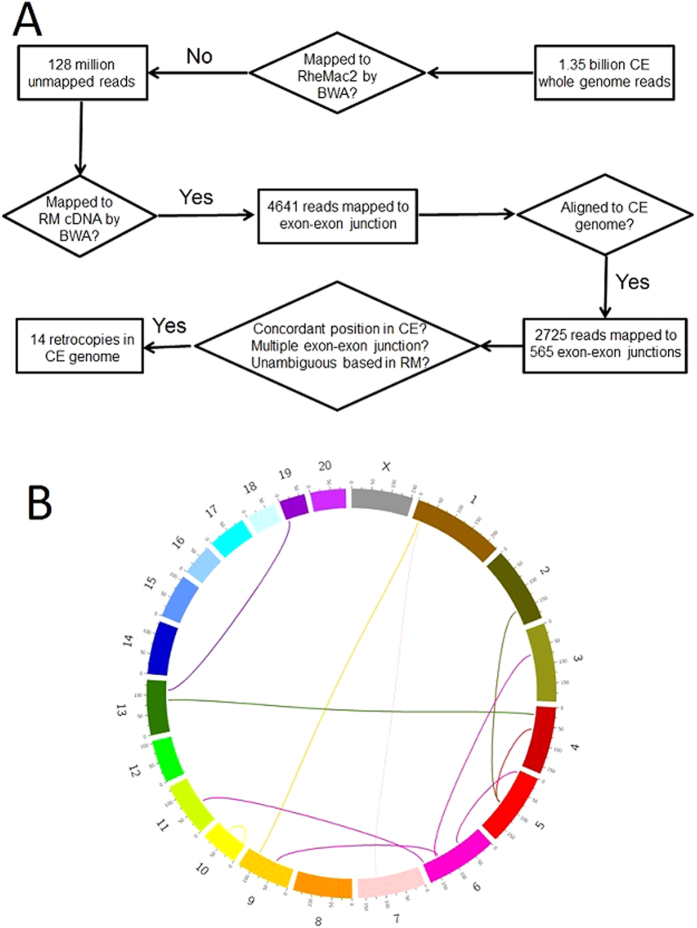
Identification of recently derived retrocopies in crab-eating monkeys. (**A**) flowchart of bioinformatics pipeline to identify candidate retrocopies using whole-genome sequencing reads from crab-eating monkey. Whole genome sequencing reads were scanned to detect those mapped to exon-exon junctions. A series of filters were applied to avoid false positives, leading to 14 highly-confidant retrocopies, half of which were further validated by PCR amplification. (**B**) The genomic distribution of retrocopies and their parental genes demonstrated by Circos plot. Chromosomes were labeled with different colors. Colored lines link retrocopies and their corresponding parental genes, the line color is consistent with the color of the chromosome on which parental genes reside.

Using BWA, we aligned 1.35 billion raw reads (~34 × genome coverage) to the reference genome of rhesus macaque (RheMac2), and found approximately 128 million reads (~9.5%) that cannot be mapped (Fig. 1A). These reads were further mapped to rhesus macaque cDNA sequences, allowing one mismatch in the seed. In total, 4,641 reads were mapped to exon-exon junctions and were labeled as potential retroposed reads. We next filtered possible RNA contaminations by removing retroposed reads failed to align to the *de novo* assembled cynomolgus monkey genome in a previous study^27^, leading to a set of 2,725 retroposed reads mapped to 565 exon-exon junctions. We defined retrocopies as genomic pieces covering two or more exon-exon junctions from the same cDNA transcript, resulting in 2,045 retroposed reads or 354 exon-exon junctions. Next, we filtered retrocopies with reads located at discordant chromosomal positions in the CE genome or partially mapped to regions flanked by ambiguous bases (Ns) in RheMac2, and obtained a final set of 14 highly-confident retrocopies in the CE genome which are absent in RheMac2 and other apes (Table 1).

### Genomic characterization of retrocopies

All of the 14 CE retrocopies were derived from 14 distinct source genes, and at least one breakpoint could be found for 11 cases, so are their exact insert positions on the RheMac2 genome. None of these 14 source genes is located on chromosome X. Similarly, of 11 retrocopies with known insert position, none is on chromosome X, and 10 were derived from inter-chromosomal retroposition (Fig. 1B). Additionally, 9 out of these 11 positioned retrocopies are in inter-genic regions, and two are in introns (Table 1). Several hallmarks are often observed for insertions derived by retroposition, such as poly-A sequences at the 3′ end or target site duplications^2^,^3^,^35^. Of 9 retrocopies with known 3′ breakpoint, poly-A sequences are presented for 7 instances. We also detected target site repeats for 3 out of 6 retrocopies with both breakpoints available (Table 1).

**Figure 2:**
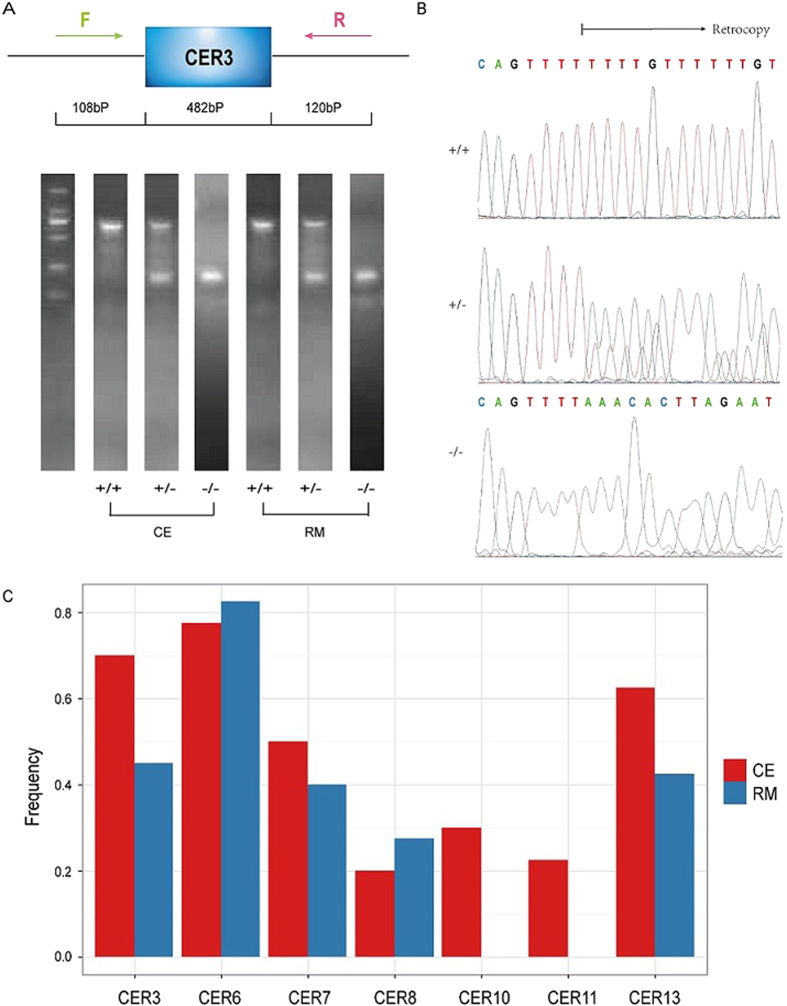
Identification of polymorphic retrocopies in macaques. (**A**) Segregating retrocopies were confirmed by polymerase chain reaction (PCR). Result from CER3 was used as an illustration. Primers were designed across the whole retrocopy insertion region (top panel), and the size of gel bands was consistent with prediction (bottom panel). The gel result was adjusted to fit the figure. CE, crab-eating monkey; RM, rhesus macaque; **+/+**, individuals with two copies of retrocopy insertion; **+/−**, individuals with two copies of retrocopy insertion; **−/−**, individuals without retrocopy insertion. (**B**) Sequencing chromatogram of individuals with two, one and zero copy of retrocopies in crab-eating monkey. The arrow denotes the breakpoint, and sequences within box in the +/− individual represent heterogeneous region. (**C**) The population frequency of retrocopies in CE and RM. Among them, CER10 and CER11 are CE-specific retrocopies.

We further experimentally validate our findings in an independent panel of 20 unrelated CE individuals by PCR, and 7 retrocopies could be confirmed, all of which are polymorphic with different frequency (Fig. 2A,C). The failure of other 7 retrocopies may suggest rare retroposition events, as the CE individual used in whole genome sequencing is not included in the validation panel. Or more likely, the quality of *de novo* assembled CE genome is less than optimal with sequencing gaps, leading to uncertainty in flanking regions of these retrocopies. Notwithstanding these possibilities, we decided to only use experimentally verified retrocopies in our downstream analyses to be conservative.

### Functional annotation of parental genes

We next sought to study functions of retrocopy source genes by using gene ontology annotations in rhesus macaque. Due to the small number of source genes, we only presented functional descriptions of them instead of performing ordinary functional enrichment analysis. Source genes of validated retrocopies are associated with 55 gene ontology terms, including 18 biological process terms, 23 cellular component terms and 14 molecular function terms (Table 2). Of them, 11 terms are found more than once, and a majority of them are associated with binding functions or membrane, which are also found in source genes in humans^10^,^33^. However, we did not see ribosomal functions enriched as in other studies^10^,^33^, probably due to the limited number of source genes.

**Figure 3:**
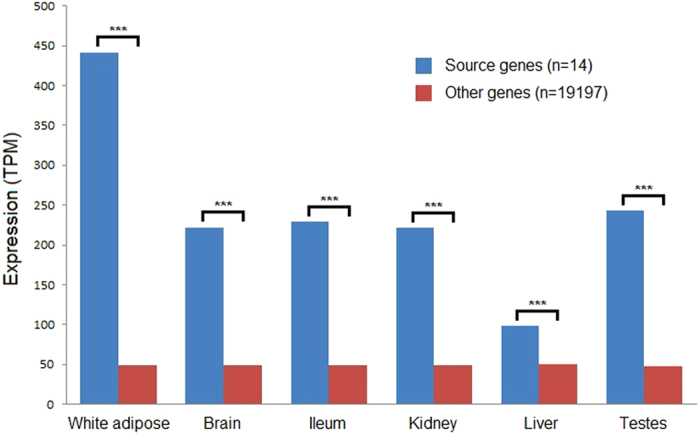
Expression pattern of parental/source genes. Compared with others, parental genes are highly expressed in multiple tissues. Gene expression was estimated by RSEM. Mann-Whitney *U* test was performed in each tissue and *** denotes a *p*-value < 0.001.

It has been argued that genes with high expression in germ cells are more likely to generate retrocopies^36^.We therefore examined the expression pattern of source genes using RNA-Seq data from six tissues. First, we estimated tissue specificity of gene expression by calculating the coefficient of variation (CV). Compared with background genes, source genes are more likely to be expressed broadly (0.47 versus 1.10, mean CV) with significance (*P* = 1.68 × 10^−5^, Mann-Whitney *U* test). In each tissue, we found that source genes are significantly over-expressed in comparison with other genes (Fig. 3), suggesting at least in CE monkeys, genes expressed at high level in multiple tissues are favored to produce retrocopies. Of note, expression estimated based on RheMac2 may be underestimated due to inter-specific differences in sequence, but it cannot explain the difference between source genes and others as we observed here, since the bias should be uniform across all CE genes. We also tried to study whether retrocopies are expressed, however, no expression could be determined due to either limited sequencing depth in the available RNA-Seq data or high sequence similarity to their parental genes, making it difficult to distinguish the origination of the mapped reads.

### Complex origination of shared retrocopies between CE and RM

CE and RM are closely related species and notable gene flow between these two species were reported by multiple studies^37^,^38^,^39^. We thus expect certain retrocopies may be shared in RM. To test this hypothesis, we performed PCR amplification for identified retrocopies in another panel of 20 unrelated RM individuals. As a result, we found five retrocopies are also polymorphic in RM with intermediate frequency (Fig. 2C), while the remaining two (CER10 and CER11) are CE-specific. To the best of our knowledge, polymorphic retrocopies shared in two species have not been reported before, despite a few studies on intra-species polymorphic retrocopies in human and drosophila^31^,^32^,^33^.

**Figure 4:**
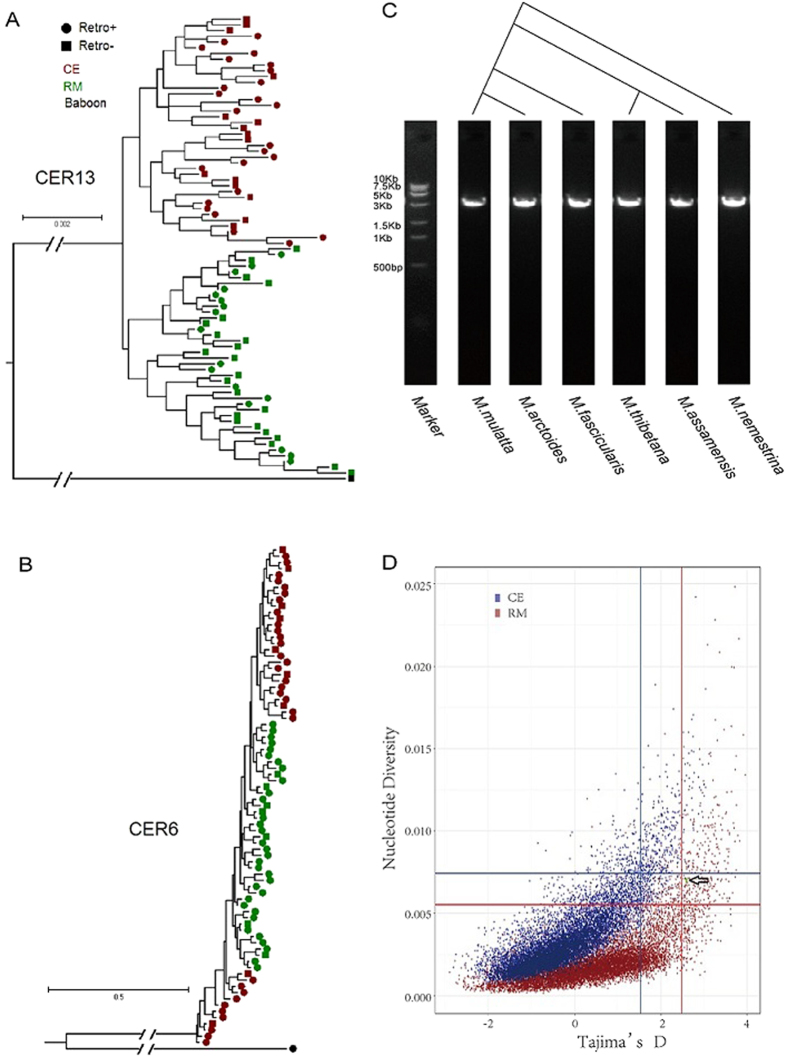
Different causes of inter-specific polymorphic retrocopies in crab-eating monkey (CE) and rhesus macaque (RM). (**A**) Phylogenetic tree of 10-kb flanking region of CER13. Solid square denotes retrocopy-absent haplotypes and solid circle denotes retrocopy-present haplotypes. CE, RM and baboon are labeled by darkred, darkgreen and black. (**B**) Phylogenetic tree of 10-kb flanking region of CER6, which is likely derived before the divergence of CE and RM and maintained polymorphic in both species. (**C**) Presence of CER6 in multiple macaques. We screened individuals from additional four macaque species and confirmed that CER6 was emerged in the common ancestor of macaques about 5 million years ago. (**D**) CER6 shows significantly high nucleotide diversity and large Tajima’s D. Both nucleotide diversity and Tajima’s D were calculated for 10,000-time coalescent simulations and were plotted. Horizontal lines represents 95 percentile for nucleotide diversity, and vertical lines represents 95 percentile for Tajima’s D. CE is colored by red, and RM is colored by blue. Green triangles denote observations in CE and RM.

Besides gene flow or hybridization between species, other evolutionary scenarios such as maintenance of ancestral polymorphisms could also explain the observation of inter-species polymorphic retrocopies. To further study the underlying evolutionary forces, we experimentally determined the allelic states (homozygous, heterozygous or absent) of each retrocopy among individuals and sequenced a 10-kb flanking region (5-kb from each side), and then derived haplotypes by fastPHASE^40^,^41^. Due to difficulties in sequencing, CER7 is excluded from downstream sequence based population level analyses. Maximum likelihood (ML) method was then applied to derive a phylogenetic tree using baboon sequence as outgroup. If the retrocopy was introduced in CE or RM after their split and later expanded to the other species, we expect that most, if not all, of retrocopy-present haplotypes in CE and RM should form a single clade. None of four inter-specific retrocopies, however, display such pattern. Instead, ancestral polymorphism in combination of different levels of gene flow fits the observed phylogenetic patterns. For example, no gene flow in the CER13 locus could be identified while substantial gene flow is found in CER3 locus (Fig. 4A and Supplementary Figure 1), and the remaining two retrocopies show intermediate gene flow (Supplementary Figure 2 and Fig. 4B).

More intriguingly, we found CER6 is presented in baboon but neither apes nor New World monkeys. Further PCR amplification also confirmed its existence in several other macaque species (Fig. 4C), including pig-tailed macaque (*Macaca nemestrina*), Tibetan macaque (*Macaca thibetana*), stump-tailed macaque (*Macaca arctoides*) and Assamese macaque (*Macaca assamensis*). This finding indicates that CER6 might be first introduced before the split of macaque and baboon approximately 10 million years ago^42^. Whether CER6 is polymorphic in baboon and other macaque species or was fixed in their common ancestors and then segregated in the ancestor of RM and CE remains unknown, and future investigation is encouraged to explore this interesting question.

Besides inter-specific polymorphisms, we also reconstructed the phylogenetic tree for CE-specific retrocopies CER10 and CER11 (Supplementary Figures 3 and 4). Despite certain level of gene flow, the phylogeny of CER11 is consistent with a recent origin in CE. The pattern for CER10 is less obvious, and the likely scenario is that CER10 is first introduced in CE, and a CER10-absent haplotype was later introgressed into RM and driven to very high frequency, if not fixed.

### Emergence age of retrocopies

Next, we tried to estimate the time scale of the emergence of retrocopies identified here. We first applied a maximum likelihood method to estimate that, assuming the generation time of 6 years and the mutation rate of 2.5 × 10^−8^ per site per generation. We found these 7 retrocopies spanned a large range from 0 to 273.5 thousand years ago (KYA, Table 3).

**Figure 5:**
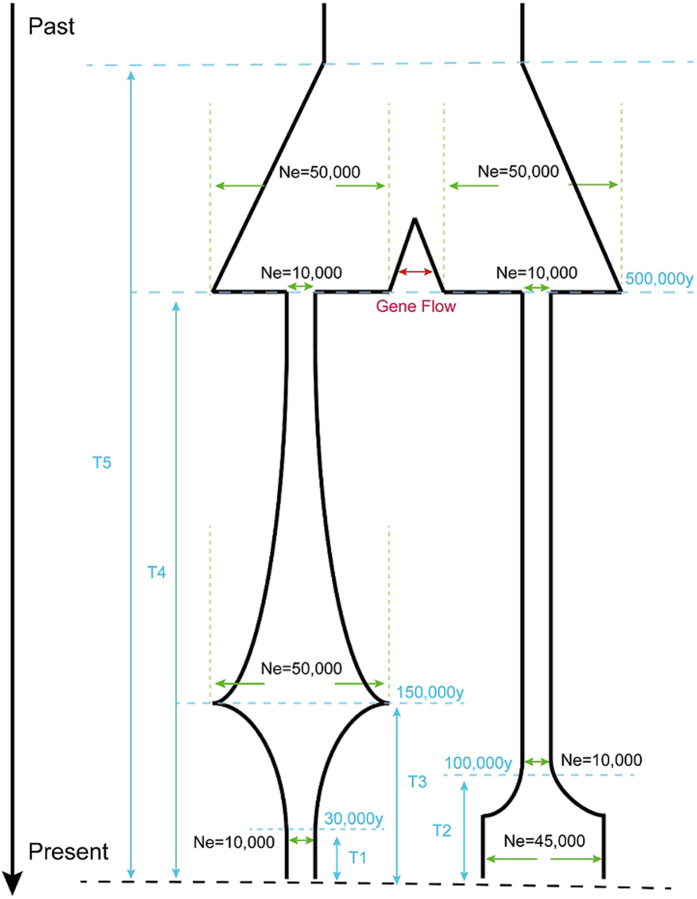
Inferred demographic models of CE and RM. Previous studies have inferred population history from individual macaque whole-genome sequences using pairwise sequentially Markovian coalescent (PSMC) model. Based on their inference, we derived the demographic history for both CE and RM. We used the inference of a Kunming-origin Chinese crab-eating monkey in our model, as both the individual used to generate whole genome sequencing data and the population panel are of Kunming origin.

Since most parental genes are evolving under functional constraints, modeling them as neutral evolution in the ML method systematically underestimates the emergence time of retrocopies. Furthermore, insertions or deletions were ignored in this model. Considering these limitations in the ML method, we also applied another method based on coalescent simulation to estimate the emergence time. First, the presence (homozygous and heterozygous) and absence states were characterized by PCR sequencing. Next we derived a plausible demographic history with gene flow (Fig. 5) based on estimates from several CE whole genome sequencing studies^22^,^38^,^43^, and performed coalescent simulation using *msms*. The median time of 5,000-run simulation for these retrocopies are from 17 KYA to 257.8 KYA (Table 3). As predicted, the coalescent-based estimate is older than ML method for all retrocopies except CER11. Notable difference was found between ML method and coalescent method for CER11, and the ML estimate is twice older than that of the higher boundary of 95% confidence interval of the coalescent estimate. Our sequencing effort shows that CER11 is restricted to CE with low to intermediate frequency (0.225), suggesting a recent emergence is more likely. Although age estimates for retrocopies presumably derived from ancestral polymorphisms are older than that of CE-specific retrocopies, it should also be noted that they are much more recent than the divergence time of CE and RM. As we will show below, balancing selection will be the likely reason to drive the discrepancy, since both models are based on the assumption of neutral evolution which may systematically underestimate the actual age.

### Evolution of retrocopies

Retrocopies are generally considered to evolve neutrally as they lack functional constraint^10^, unless they maintain important functions and could be subject to purifying selection by showing a low evolutionary pace. To characterize the possible evolutionary scenario, we compared genomic sequences of retrocopies with their parental genes in a comparative genomics framework. Of 7 retrocopies, multiple frameshift indels were observed in 4 when aligning with coding sequences of parents, implying these retrocopies may be non-protein coding and free to accumulate disruptive mutations. For retrocopies with intact open reading frames, we first estimated their evolutionary rate using “free-ratio” model in *PAML*. Neither non-synonymous nor synonymous changes could be identified in CER8 and CER13. For the remaining retrocopy CER10, *ω* < 1 was observed, a sign of possible purifying selection. We next compared the alternative “two-ratio” model to the null “two-ratio” model with a fixed *ω* = 1, but no significant result was achieved, suggesting CER10 may evolve neutrally. Therefore, no evidence could be found to support that these retrocopies have protein-coding properties.

Since CER6 has been polymorphic for several million years in different macaque species and is also presented in baboon, it is of great interest to understand the evolutionary forces that shape this pattern. Balancing selection is one way to maintaining ancestral polymorphisms, leading to a higher nucleotide diversity (*π*) and elevated *Tajima’s D* than expected under neutral evolution^44^,^45^,^46^. To test this, we first calculated both *π* and *D* statistics for a 10-kb flanking region of CER6 in CE and RM, and found large values of *π* (0.007 for both species) and *Tajima’s D* (2.592 and 2.595 for CE and RM, respectively). We next performed 10,000-time coalescent simulations using deduced demographic model for CE and RM, and found that the observed *π* and *D* statistics are generally greater than those derived from neutral model, in other words, the observed pattern could not be explained by demographic history (all *P* < 0.05 except for *π* in CE, where *P* < 0.1, Fig. 4D and Table 4), suggesting balancing selection may be the major force to maintain CER6 in different macaque species.

We also investigated the pattern of other five retrocopies by coalescent simulation (Table 4), but no elevated nucleotide diversity could be detected except for CER3 in CE (*P* = 0.048). Additionally, CER11 demonstrated an extremely high *Tajima’s D* (3.45, *P* = 0.004), a strong signal of balancing selection. Similar pattern is also found in RM (*Tajima’s D* = 2.69, *P* = 0.003). However, we noticed that CER 11 is a CE-specific retrocopy, making it less likely to be the target of balancing selection. Since the nearest gene of CER11 is more than 200 kb away, it is possible the true target of selection may be non-coding elements within or near this 10-kb region. Further effort is encouraged to identify the actual target, though it is beyond the scope of this study. High *Tajima’s D* values were also observed for two other retrocopies in RM (CER8 and CER13), further indicating balancing selection could play a role in maintaining ancestral polymorphic retrocopies across species.

## Discussion

Diversity in DNA sequences is the foundation of evolution. As a major mechanism in genome evolution, retroposition introduces genomic variation by integrating mRNA transcripts back into host genomes. Previous studies highlighted inter-specific comparisons of retrocopies that were derived long time ago and fixed within species, demonstrating their contribution to adaptive evolution by introducing new genes with distinct functions^3^,^35^. The emergence of new sequencing technologies substantially reduces the cost of whole genome sequencing and makes it also possible to characterize genomic changes at the population level. Several recent studies revealed segregating retrocopies in a number of human populations^32^,^33^,^47^,^48^, which may play an important role in human adaptation and phenotypic evolution. However, a majority of the studies were focusing on humans and a few model animals such as mouse or drosophila, efforts in other primates such as macaque, an important model animal widely used in biomedical studies, are still scares^10^,^49^.

In this study, we presented the first effort to identify young retrocopies in the CE genome and profiled their evolutionary pattern. Of 14 retrocopies detected, 7 were also confirmed in an independent validation panel, all of which are macaque-specific except CER6 and are segregating in CE. Surprisingly, we found five retrocopies are also polymorphic in RM despite their split from CE several million years ago. Further analysis suggests the observed inter-specific polymorphic retrocopies emerge as the result of ancestral polymorphism and gene flow between these two species further complicates their phylogenetic pattern. The signal of balancing selection was found for a few retrocopies, implying their potential role in evolutionary adaptation.

It should be noted that the whole genome sequencing data is from only one CE individual, it thus inevitably underestimates the total number of polymorphic retrocopies, as certain incidences may not be presented in the particular individual. We also required that a candidate retrocopy should include two exon-exon junctions, and its sequence must be presented in the *de novo* assembled CE genome. Since there are a number of gaps in the assembled CE genome, these filters were conservative and could miss some cases. Moreover, our strategy ignores retrocopies that are presented in the RM reference genome but remain segregating within and among species. Therefore, the list of retrocopies we identified here only presents the minimal set of young retrocopies in CE, and whole genome sequencings from additional individuals or improved annotations will definitely supplement the current catalogue.

Retroposition could have functional consequence via various routes. One way of particular interest is that the new retrocopy was specifically expressed to interplay with its parental gene or was integrated into the coding regions of existing genes^2^,^35^. However, genomic and transcriptomic analyses suggest none of retrocopies identified here fell into this category. Retrocopies could also obtain functionality when they were inserted into regulatory regions of host genes. In fact, we found two retrocopies are in the intronic region of protein-coding genes. One gene is *STPG1*, which was reported in humans to be involved in the induction of apoptosis^50^. There were another two retrocopies located within 2 kb of genes, and could regulate the expression of their flanking genes. We thus encourage future studies to characterize the consequence of those interesting retrocopy insertions.

Although segregating retrocopies have been identified recently in a few number of species, this is the first time to our knowledge to observe inter-specific retrocopies in closely related species which are still segregating. As well appreciated, gene flow between CE and RM are not uncommon. Inter-specific hybridization and gene flow have been reported between rhesus macaques and crab-eating macaques in various datasets, including morphology, mtDNA, Y-chromosome, short tandem repeats (STRs) and single nucleotide polymorphisms (SNPs), and introgression of rhesus macaque DNA into crab-eating macaque genome is the dominant direction of gene flow^37^,^51^,^52^,^53^,^54^,^55^,^56^. It is possible that a retrocopy could be derived after the split of CE and RM and then become polymorphic in both species through gene flow. However, our sequencing analysis provides no support for this scenario. The most likely explanation is these segregating retrocopies are descendant from ancestral polymorphisms, where they are polymorphic in the common ancestor of CE and RM and were passed to each species after divergence. Under the scenario of neutral evolution, it is extremely unlikely a segregating mutation could be maintained for millions of years. Indeed for the ancestral polymorphism CER6, we found significantly elevated *Tajima’s D* and nucleotide diversity in both CE and RM, a signature of balancing selection. Balancing selection is an evolutionary force that persists genetic variations within a population for an extended period, targets of balancing selection includes the sickle cell hemoglobin polymorphism in humans^57^, genetic variation in the long to middle wavelength-sensitive (L-M or red-green) opsin gene in New World monkeys^58^, and R-gene loci in Arabidopsis^59^. Recently, Leffler *et al*. identified six cases of non-coding long-lived ancient balanced polymorphisms that persisted to the present in humans and chimpanzees^44^, and suggested the targets may be involved in host-pathogen interaction. Interestingly, CER6 is approximately 1 kb away from ENSMMUG00000030652, a possible immunoglobulin gene in macaques. It is possible that this gene is the actual target of balancing selection and the pattern for CER6 is a hitchhiking effect. However, the functional and evolutionary relevance of ENSMMUG00000030652 is still unknown. CER6 may interplay with this gene to participate in the host-pathogen interaction, which can be tested in future studies.

In conclusion, our study reports the first observation of inter-specific segregating retrocopies in two closely related macaque species. Our analysis suggests maintenance of ancestral polymorphism is the main cause to generate this pattern. Balancing selection is also identified to act on at least one retrocopy, which may be involved in the host-pathogen interaction. Since genome sequences from only one CE individual was used in our study, we thus believe recruiting more macaque samples could further improve our understanding how retroposition shapes genome evolution in macaques and other primates.

## Materials and Methods

### Identification of retrocopies

Whole genome deep sequencing reads of a cynomolgus monkey^27^ were downloaded from NCBI short read archive (SRA), using the accession number SRP006913. Only 75-bp pair-ended reads were used in this study to avoid potential false positives caused by short read length, resulting in 1.35 billion raw reads or ~34X genome coverage (calculated based on the rhesus macaque genome size^26^). To identify recently emerged retrocopies, we developed a stringent bioinformatics framework with high sequence similarity requirement. Reads were first mapped to the reference genome of rhesus macaque (RheMac2) by Burrows-Wheeler Aligner (BWA)^60^, allowing one mismatch in the seed (Fig. 1). After alignment, we collected unmapped reads and their paired reads to perform another round of mapping to rhesus macaque cDNAs. A read was denoted as potential retroposed read if it fulfills all of the following criteria: 1) there are at most five mismatches in the alignment; 2) the read covers one exon-exon junction and 10-bp flanking sequences on both sides; 3) there is at most one mismatch within the 20-bp region centering in the exon-exon junction. To further remove potential impact of RNA contaminations in genomic DNAs, we also mapped by BLAT retroposed reads to *de novo* assembled CE genome published before (http://gigadb.org/dataset/100003), and filtered reads with <95% sequence similarity and <95% read length coverage. Next, we required that a candidate retrocopy must contain retroposed reads covering two or more exon-exon junctions and all of the junctions should be located on appropriate chromosomal positions in the CE genome. Based on the mapping position of retroposed reads, we also extracted sequences of candidate CE retrocopies as well as a 500-bp flanking region on both sides and mapped them back to RheMac2 to remove cases that have highly similar intron-free alignments (>90% sequence similarity and >90% sequence length coverage) or were partially aligned to regions flanking with ambiguous bases (Ns).

### PCR validation of retrocopies

First, we used TIANamp Genomic DNA kit (TIANGEN Biotech, Beijing) to extract genomic DNA of blood samples from 20 rhesus macaque individuals bred in Kunming Institute of Zoology and 20 wild crab-eating monkey individuals following recommended protocols. Then primers spanning exon-exon junctions were designed using Primer5 software (http://www.premierbiosoft.com/primerdesign/index.html) to confirm the presence and inserted position of retroposition (Supplementary Files). Regular PCR was performed using TAKARA Ex-Taq polymerase and LA-Taq polymerase in 25 ul reaction volume with template DNA (10 ng/ul). By looking for bands consistent with predicted size, we confirmed the presence of retroposition. We further carried out G-50 purification on Resin-purified PCR products and Sanger sequencing following the recommended protocol to confirm the presence of retroposition ultimately. For one retrocopy (CER6), we also sequenced one individual from four additional macaque species: pig-tailed macaque (*Macaca nemestrina*), Tibetan macaque (*Macaca thibetana*), stump-tailed macaque (*Macaca arctoides*) and Assamese macaque (*Macaca assamensis*).

### Sequencing of flanking regions

We first applied long-range PCR to amplify the 10-kb flanking region of retrocopies of known insertion site, The primers were designed using Primer5 (http://www.premierbiosoft.com/primerdesign/index.html), and the amplification was performed using LA-Taq Polymerase(TAKARA) in 50 μl reaction volume with template DNA (10 ng/μl). PCR products were then Resin-purified and sequenced using BigDye terminator sequencing kit (ABI) on an ABI 3730 automated sequencer. All primers are available in Supplementary Information.

### Reconstruction of phylogenetic trees

To reconstruct the phylogenetic tree for retrocopy loci, we experimentally determined the allelic state (homozygous, heterozygous or absent) of retrocopies. This information is aggregated with flanking sequences to generate haplotypes using fastPHASE^40^,^41^. Then we used MEGA 6.0^61^ to construct maximum likelihood tree using General Time Reversible (GTR) model and Gamma distributed rate variation among sites. We also tested uniform rate and obtained similar results. The pre-released baboon sequence was downloaded as outgroup. Sequence alignments are available upon request and inferred trees in newick format could be found in Supplementary Information.

### Age estimation of retrocopies

We first used a maximum likelihood method to estimate the emergence time of retrocopies, which is modified from Mendez *et al*.^62^. We assume that the mutation rate is known and that no gene conversion occurred between parental genes and retrocopies. *K*_*1*_ and *K*_*2*_arethe number of mutations for the parental gene and the retrocopy since the retroposition event occurred *t* years ago, *μ* is the mutation rate (per year per site) and *l* is the number of sites that a retrocopy could be aligned to its parental gene, the likelihood function for *t* can be written as


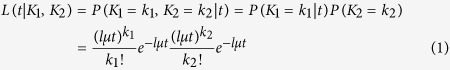


By solving the function


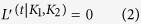


We obtained the maximum likelihood estimate as


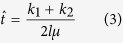


The common ancestor of retrocopy and its parental gene was reconstructed by *baseml* embedded in *PAML*, using human sequence as outgroup to avoid bias in gene tree phylogeny. A generation time of 6 years and a mutation rate of 2.5 × 10^−8^ per site per generation for macaque were applied in our calculation^38^.

For a subset of retrocopies subject to sequencing later at the population level, we also used coalescent simulation to estimate their emergence time. Based on inferred population history from individual macaque whole-genome sequences using pairwise sequentially Markovian coalescent (PSMC) model^22^,^27^,^38^,^63^, we derived a likely demographic history for both CE and RM, and then used the *msms* program^64^ to perform the simulation. For a given retrocopy, we simulated coalescent trees based on the demographic model we derived based on previous studies, using 10,000 as the current effective population of CE, a generation time of 6 years and a mutation rate of 2.5 × 10^−8^ per site per generation as above. Since haplotypes with the retrocopy insertion should coalesce first, we examined trees and removed ones that are not consistent with the observed number of retrocopies in CE and RM, respectively. This process has been repeated until we have 5,000 qualified trees. We then used the simulated coalescent time of retrocopies as the estimate age of retrocopies. The *msms* command used to generate trees is “java -jar msms3.2rc-b163.jar -N 10000 -ms 80 5000000 -I 2 40 40 -T -n 2 4.5 -m 21 1.92244 -m 120.55646 -eg 0.125 1 −3.218876 -eg 0.625 1 1.103615 -en 2.083333 1 5 -eg 0.125 2 5.156836 -eg 0.4166667 2 0.0 -en 2.083333 2 5 -ej 15.41667 1 2”.

### Evolutionary analysis of retrocopies

We first retrieved orthologs of parental genes in other primates (human, chimpanzee, gorilla, orangutan, gibbon, baboon, and marmoset) using BLAT incorporated in UCSC genome browser^65^. Then retrocopies, corresponding parental genes and orthologs were aligned based on codons using MUSCLE^66^. To estimate the ratio of non-synonymous versus synonymous substitution rates (*d*_*N*_*/d*_*S*_ or *ω*) of each branch, “free-ratio” model in *codeml* embedded in PAML was applied^67^. To test rapid evolution in retrocopies, we compared two “two-ratio” models: in the null model, parental genes and their orthologs have one common *ω* value while retrocopies *ω* value fixed as 1; in the alternative model, *ω* of retrocopies was estimated. The statistical significance was measured by the likelihood ratio test (LRT) to determine whether retrocopies have the *ω* value significantly different from one.

We also used *DnaSP*^68^ to perform population genetics analyses including calculating nucleotide diversity and *Tajima’s D*. Since CE and RM have undergone a series of demographic events, we used coalescent simulation to assess the statistical significance of the observed nucleotide diversity and *Tajima’s D*. A total of 10,000 simulations were generated by *msms* using “java -jar msms3.2rc-b163.jar -N *N*_*e*_ -ms 40 10000 -I 1 40 -t 4*N*_*e*_*μl* -eg 0.125 1 −3.218876 -eg 0.625 1 1.103615 -en 2.083333 1 5” for CE and “java -jar msms3.2rc-b163.jar -N *N*_*e*_ -ms 40 10000 -I 1 40 -t 4*N*_*e*_*μl* -eg 0.02777778 1 5.156836 -eg 0.09259259 1 0.0 -en 0.462963 1 1.1111” for RM, where *N*_*e*_ is 10,000 for CE and 45,000 for RM, *μ* is 2.5 × 10^−8^ per site per generation, and *l* is the size of actually sequenced locus.

### Gene expression analysis of parental genes

RNA-Seq data was used to estimate the expression abundance of genes in CE monkeys^27^. Transcriptome sequencing data from CE brain, ileum, kidney, liver, testis and white adipose were retrieved from NCBI SRA with accession number GSE29629. Since CE monkeys and rhesus monkeys diverged recently and have a low sequence divergence rate (0.40%)^27^, we used the well annotated RheMac2 genome instead of the assembled CE genome^27^ to avoid bias caused by incomplete annotation, and estimated gene expression by RSEM^69^, using the Ensembl^70^ protein-coding annotation file (release 69).

To investigate tissue expression pattern, we calculated the coefficient of variation (CV) of expression for each gene, which is the ratio of standard deviation to mean. Genes with small CV are expressed in a wide range of tissues and vice versa.

### Functional annotation and analysis of parental genes

To annotate parental genes in CE monkeys, we used the gene ontology (GO) information^71^ from their rhesus monkey orthologs, which we retrieved using Ensembl (release 69). Functional enrichment tests for these genes were performed against all other genes with rhesus monkey orthologs by TopGO^72^, an Rpackage that uses Fisher’s exact test to compare the difference in occurrences in a given functional category between foreground and background sets. Multiple test correction was performed using the Benjamini-Hochberg approach^73^.

### Statistical analysis and plot

Unless specified, all statistical analyses were performed using R (https://www.R-project.org). Plots were generated using R package ggplot2 (http://had.co.nz/ggplot2). Circos plot was drawn using Circos^74^.

## Additional Information

**How to cite this article**: Zhang, X. *et al*. Emergence and evolution of inter-specific segregating retrocopies in cynomolgus monkey (*Macaca fascicularis*) and rhesus macaque (*Macaca mulatta*). *Sci. Rep.* **6**, 32598; doi: 10.1038/srep32598 (2016).

## Supplementary Material

Supplementary Information

## Figures and Tables

**Table 1:** Summary information for recent retrocopies identified in CE.

ID	Length (bp)	#Exons	Source Gene ID^a^	Source Gene Symbol	#Source Gene Exons	Source Gene Position^b^	Insert Position	Flanking Gene ID	Flanking Gene Symbol	Distance	RheMac3	Target Site Repeat	Poly-A
CER1	1071	5	ENSMMUG00000005755		9	chr10:58149770–58177848	chr10:10178266	ENSMMUG00000017300		79520	No	AG	No
CER2	489	5	ENSMMUG00000013163	*COPG1*	*28*	chr11:85564900–85586291	NA^c^				No	NA	NA
CER3	290	3	ENSMMUG00000003444		3	chr13:85457959–85458977	chr4:23276507	ENSMMUG00000032944		135849	Yes	NA	Yes
CER4	846	4	ENSMMUG00000016540		6	chr14:83953614–83960436	NA				No	NA	NA
CER5	424	3	ENSMMUG00000016665		5	chr19:5796828–5805003	NA				No	NA	NA
CER6	1353	11	ENSMMUG00000007904		13	chr19:6284015–6297962	chr13:113137936	ENSMMUG00000030652		1065	Yes	No	Yes
CER7	2755	8	ENSMMUG00000003048		8	chr2:148471045–148483966	chr5:146612859	ENSMMUG00000017834		14701	Yes	No	Yes
CER8	986	4	ENSMMUG00000008177		4	chr5:102625400–102636770	chr4:64381666	ENSMMUG00000029593		Intronic	No	GAAAATGGAAGATTTA	Yes
CER9	1426	8	ENSMMUG00000012463		24	chr6:131186415–131261513	chr9:50073946	ENSMMUG00000007562		28074	No	NA	Yes
CER10	424	3	ENSMMUG00000010011		3	chr6:137046689–137048723	chr3:70012370	ENSMMUG00000030369	*NUPR1L*	80	No	AAAAGGTC	Yes
CER11	357	5	ENSMMUG00000010843	*HNRNPH1*	11	chr6:176246196–176256472	chr11:58680347	ENSMMUG00000011579	*FAM19A2*	246385	No	NA	NA
CER12	707	4	ENSMMUG00000009140		5	chr6:67217608–67238670	chr5:7020592	ENSMMUG00000036342		132119	No	No	No
CER13	843	5	ENSMMUG00000012325		5	chr7:117432932–117447846	chr1:27048728	ENSMMUG00000004464	*STPG1*	Intronic	No	NA	NA
CER14	377	4	ENSMMUG00000014037		5	chr9:103079065–103086813	chr1:18897328	ENSMMUG00000018243		27155	No	NA	Yes

^a^Rhesus monkey gene IDs are used, as the crab-eating monkey genome is not well annotated.

^b^The genomic position is based on the assembled genome crab-eating monkey. We also examined the corresponding position in rhesus monkey (RheMac2) to assure consistency.

^c^NA indicates that retrocopy is mapped on scaffolds and its chromosomal identity is not available.

**Table 2:** GO terms shared in multiple source genes.

ID	Term	#Gene	Category^a^
GO:0016020	membrane	5	CC
GO:0005634	nucleus	4	CC
GO:0005739	mitochondrion	4	CC
GO:0000166	nucleotide binding	3	MF
GO:0005737	cytoplasm	3	CC
GO:0003676	nucleic acid binding	2	MF
GO:0003677	DNA binding	2	MF
GO:0005515	protein binding	2	MF
GO:0005524	ATP binding	2	MF
GO:0005622	intracellular	2	CC
GO:0005743	mitochondrial inner membrane	2	CC
GO:0005794	Golgi apparatus	2	CC
GO:0016021	integral to membrane	2	CC

^a^Categories defined in gene ontology. CC, cellular component; MF, molecular function.

**Table 3:** Retrocopy age estimate by ML and Coalescent Simulation.

ID	Length (bp)	K_retro^a^	K_parent^b^	AgeML (ky)^c^	AgeCS (ky)^d^	(95% C.I.)
CER1	1071	12	2	43.6	—	—
CER2	489	20	4	163.6	—	—
CER3	290	5	0	57.5	257.8	(79.3, 678.5)
CER4	846	24	17	161.5	—	—
CER5	424	11	7	141.5	—	—
CER6	1353	84	27	273.5	405	(163.3, 1046.0)
CER7	2755	62	24	104.1	—	—
CER8	986	2	2	13.5	152	(44.7, 413.5)
CER9	1426	20	4	56.1	—	—
CER10	424	0	1	7.9	21.8	(6.8, 55.8)
CER11	357	3	9	112.0	17	(4.4, 45.9)
CER12	707	1	0	4.7	—	—
CER13	843	0	0	0.0	243.9	(74.5, 571.1)
CER14	377	6	3	79.6	—	—

^a^Estimated nucleotide substitution in retrocopy.

^b^Estimated nucleotide substitution in parental gene.

^c^Age estimated using maximum likelihood method.

^d^Age estimated using (5,000-run) coalescent simulation method for retrocopies which are successfully sequenced.

^e^95% confidence interval of coalescence-based age estimate.

**Table 4:** Summary statistics of population genetics analysis in CE and RM.

ID^a^	Size	Segregating Sites		Nucleotide Diversity^b^		Tajima’s D^b^	
		CE	RM	CE	RM	CE	RM
CER3	10078	183	181	0.006*	0.006	1.33	1.36
CER6	10062	178	174	0.007*	0.007	2.59*	2.59**
CER8	9969	85	88	0.003	0.003	1.77	2.38**
CER10	10141	54	43	0.002	0.002	2.12	3.08***
CER11	10044	58	68	0.003	0.003	3.45**	2.69**
CER13	9922	55	45	0.002	0.002	1.85	2.91**

^a^CER7 is excluded in the list because the sequencing effort is failed.

^b^Significance: *p < 0.05, **p < 0.01, ***p < 0.001.
