# Supplementary material for: Emergence and evolution of inter-specific segregating retrocopies in cynomolgus monkey (Macaca fascicularis) and rhesus macaque (Macaca mulatta)
Source: Sci Rep. 2016 Sep 7;6:32598. doi: 10.1038/srep32598 (PMC5013489; doi:10.1038/srep32598)

**Emergence and evolution of inter-specific segregating retrocopies in cynomolgus monkey (*Macaca fascicularis*) and rhesus macaque (*Macaca mulatta*)**

**Xu Zhang^1,3#^, Qu Zhang^2,4#*^, Bing Su^1*^**

^1^ State Key Laboratory of Genetic Resources and Evolution, Kunming Institute of Zoology, Chinese Academy of Sciences, Kunming 650223, China

^2^ Department of Human Evolutionary Biology, Graduate School of Art and Science, Harvard University, Cambridge, MA 02138, U.S.A.

^3^ Kunming College of Life Science, University of Chinese Academy of Sciences, Beijing 100049, China

^4^ Perspective Sciences, 11 Xinghai Avenue, Shenzhen 518054, China

**^#^ These authors contributed equally to this study.**

**Running Title**: Inter-specific segregating retrocopies in macaques

**^*^Correspondence to**: [sub@mail.kiz.ac.cn](mailto:sub@mail.kiz.ac.cn) or [quzhang@post.harvard.edu](mailto:quzhang@post.harvard.edu)

Supplementary Table 1 Confirmation PCR primers

| CER11_S | TGGATTGGCAGAGCAACATA |
| --- | --- |
| CER11_R | AGCTGCTCAATCCTGTGAGAG |
|  |  |
| CER13_S | TAATTAGATACTGTTTCAATGAAACCA |
| CER13_R | GGCCCTGTCTGTACAAAAATAATAA |
|  |  |
| CER8_S | TTAAGGCGATACACCCTCTCA |
| CER8_R | TTCAACCAAAGCCCATCCTA |
|  |  |
| CER10_S | TTAAAAGAGAGCAGCAAGAATGG |
| CER10_R | CTGGGATTACAGGAGTGAACTACTT |
|  |  |
| CER6_S | AGAAACATAGAACACCTCCACCAAATGGAT |
| CER6_R | TGTCCCCTTAAATCTACCTGCTGTTACCTT |
|  |  |
| CER3_S | ATGTTTCTTTGTTTTGAGTCAGGT |
| CER3_R | CTTCTCTCTGGATAGTAAGCCTCC |

Supplementary Table 2 Long range PCR primers

| CER11_LA_S1 | TAGGATCCCGTTCTAAACATAAAAGTCATTTGT |
| --- | --- |
| CER11_LA_R1 | GACCAGTGTTACACTCTCAATTCAATCTGGTT |
| CER11_LA_S2 | GTCTGTAAACCAGATTGAATTGAGAGTGTAACAC |
| CER11_LA_R2 | ATCAACTGTCCAAGGAATGTTACATACTGTTAAC |
| CER11_LA_S3 | GAGAAAACCTCATAAGAGGAGATCACTTGATG |
| CER11_LA_R3 | ACCTCTACCTCATGAGTTCAAGCTATTCTGC |
|  |  |
| CER13_LA_S1 | TTTCAATTCTTTGTGTAAATCCAAGATTCTTTCT |
| CER13_LA_R1 | TTACTGTCAATCAGACTGTTTCCGTTCATATCT |
| CER13_LA_S2 | TTGTCTCAGTGCTTGTGTTCAAGTAACTCTTACT |
| CER13_LA_R2 | CCTCTAATGATTGGTCAATAACTTTTCACGTTA |
| CER13_LA_S3 | GTTTCTTTAACGTGAAAAGTTATTGACCAATCA |
| CER13_LA_R3 | CTCTTTCCGAACAAATCAATAAAACACAGTC |
|  |  |
| CER8_LA_S1 | CAAGTGAGAATGCAAATTTATTCAGACACTTTT |
| CER8_LA_R1 | TTACCATGCAATACAAATGTCCTCACGTATAC |
| CER8_LA_S2 | ATTGTAATAATAAGAAGTAAAGTCCAAGCGACAGC |
| CER8_LA_R2 | CCTGAAATACAAGTCAGTGAAAAGGAAAGTAATTAG |
| CER8_LA_S3 | ATTACCCAGTCTGTGATATTCTGATCTAGTAGCAC |
| CER8_LA_R3 | TCGGGGAGACTTAAAGAGTAAATATATGTGACC |
| CER8_LA_S4 | GCAAATATAGAATGGATAGAAGATAAGAAAGTGGA |
| CER8_LA_R4 | ATTGAACTAATGGAGATAAAGAATAGAAGGAGGGT |
| CER8_LA_S5 | CACATATATTTACTCTTTAAGTCTCCCCGAAATG |
| CER8_LA_R5 | TTGTTCAGTGTACTTGTATCTCCCATTAGTGTACA |
|  |  |
| CER10_LA_S1 | TCTCTCCTTGTAAGGTGCTTTGTAGTATCTGTCT |
| CER10_LA_R1 | GGTTTATGTTGGTCAAAAATAGGAAACTATGTTC |
| CER10_LA_S2 | CAGTGACCTGAAATTATTGACTGACTATTCCA |
| CER10_LA_R2 | GTCTCTCTCTGCTTATCTTTGTGACCTTTTCT |
| CER10_LA_S3 | GATACCGAAGGACCTGTTCAAAGTATTTTCT |
| CER10_LA_R3 | GCAAACTACATCATAAAAGGTACTGTGGCTT |
| CER10_LA_S4 | TTCATATATAGTTTTATCCAGAATTGTCCTGTTGG |
| CER10_LA_R4 | ATGCATAAAATATCTGAAGTTGATAACACTGGTT |
|  |  |
| CER6_LA_S1 | GTCCCTGAGTTAAATGCATAGGCTACATTT |
| CER6_LA_R1 | AGTGGGTAATGACAAGAGGAGATGTATACACA |
| CER6_LA_S2 | CATTCTCTAGGTTGCCTATTCACTCTGATGATA |
| CER6_LA_R2 | CCCTTTAACAGTTTTGAAAACCTCTGTTCAC |
| CER6_LA_S3 | TGAAGATGTTGCAACTTATTACTGTCAACAGG |
| CER6_LA_R3 | ACCTATCTAGAGAGGCCAAAATTCAAATTCA |
|  |  |
| CER3_LA_S1 | TTGCAATAATTGCTGCTATCAGAAATAAGCT |
| CER3_LA_R1 | CAGAAAACAGGCATTTCTCTTCTAACTCTCTAA |
| CER3_LA_S2 | CTTGTACACAGAGGAAGTTATCCTGAATTGG |
| CER3_LA_R2 | CACCAAAATATTCTTGACTGCAAAATAACAGA |
| CER3_LA_S3 | GTCACTAGAGGAATTTAAGCAGAGGAGAGACA |
| CER3_LA_R3 | CTGAATTACGTCCCTTCAAAATGTATATGTTG |

Supplementary Table 3 Sequencing primers

| CER11_Seq_S1 | ATGTCAGCTCTCAAAGAGTTTCA |
| --- | --- |
| CER11_Seq_S2 | ACATTTAGGCTCTGAGTGAATTGT |
| CER11_Seq_S3 | AACCTAGGATGTTATGTGTTCACAG |
| CER11_Seq_S4 | CTGAGACAACATTGCTAACAAACTC |
| CER11_Seq_S5 | TTTAAGTTGTAGGGTACATGTGCA |
| CER11_Seq_S6 | TTTCTTAATCCAATCTGTCACTGAT |
| CER11_Seq_S7 | CCTTCTAGTAATCCTCAGTGCCTAT |
| CER11_Seq_S8 | TGATGTTCTGTTTTAAGGTCTTCAA |
| CER11_Seq_S9 | CTTCACTTTAATTAGGTCCCTCTAA |
| CER11_Seq_S10 | CTGGAATAGAATACTGCATGTGTGT |
| CER11_Seq_S11 | ACACATGACTGACTGCTACTGTGA |
| CER11_Seq_S12 | GGTTGATTGTATGAGCACTTCTAAG |
| CER11_Seq_S13 | GAGAGGGTAGGTTTAATCAGTGTTC |
| CER11_Seq_S14 | TTTCATAAAGGATTGTGTTCTGATC |
| CER11_Seq_S15 | TTGTAGAATCATCCTGAGTGTGTTT |
| CER11_Seq_S16 | AGTATGATGCTCATAAGTGTGTGCT |
| CER11_Seq_S17 | ATCACCTAAGCTGCTTGTAATAATG |
| CER11_Seq_S18 | AATGTTTCCAGAGTGATCCAGAA |
| CER11_Seq_S19 | AAACTGTTCATCTCACTAAACAAGC |
| CER11_Seq_S20 | ATGAGAAAATATTTCCTCCAAGTC |
| CER11_Seq_S21 | ACAAACGATAGACCTGATTCTGAG |
| CER11_Seq_S22 | TATGTGGAATGGCTTAAGAAAAGTA |
| CER11_Seq_S23 | GACATATACAGCTCCCTATTGCTG |
| CER11_Seq_S24 | GGCTACAGCTGTTTCTAAGTCAAA |
| CER11_Seq_S25 | GATTTATTAGCATCTTGCCATTTTA |
| CER11_Seq_R1 | TTATGTACTCTTTGGGCAATTTG |
| CER11_Seq_R2 | TCCATCATTCTTAGCAAACTATCAC |
| CER11_Seq_R3 | ATAAATGAATGGAGCTTGTTTAGTG |
| CER11_Seq_R4 | TCCTAAATTCTTATTTACAAAGCCC |
|  |  |
| CER13_Seq_S1 | CTTGGCATAATTTTCTTTATGTCTT |
| CER13_Seq_S2 | TGATCTTCTGAAGTCTCTACTCAGC |
| CER13_Seq_S3 | TGAACTCTACCCAATTCCTATCTAA |
| CER13_Seq_S4 | TGTCCATTGTGAGTTGTTTAACATA |
| CER13_Seq_S5 | AAACAACAGAAACGAGTCAATAATG |
| CER13_Seq_S6 | CCCTAGCTTTAATAAAGCTGTTATT |
| CER13_Seq_S7 | CCTTTAAGTGAAAAGGTGAACGT |
| CER13_Seq_S8 | ACGGAAACAGTCTGATTGACAGTA |
| CER13_Seq_S9 | CAAAACAAACAAACAGACAAATAAA |
| CER13_Seq_S10 | CACAGGGATATCAAATTAACAACTT |
| CER13_Seq_S11 | TCATAGAGACAATTTCTCACTATGC |
| CER13_Seq_S12 | TCCTAATTCCTACCCTTGGAAT |
| CER13_Seq_S13 | TAAGCAGCACTAACCAATCTCTCT |
| CER13_Seq_S14 | AATACTAGAGGTAGACCCTTTTTGG |
| CER13_Seq_S15 | GTTCCAATCTCACCCTTTTATTT |
| CER13_Seq_S16 | CTTTAACGTGAAAAGTTATTGACCA |
| CER13_Seq_S17 | TAGATTGACATAGGCGACTATACAA |
| CER13_Seq_S18 | TCCCTCTTTCTCATTGTAGATGTCT |
| CER13_Seq_S19 | TCAAGGACGTTTTAGATTTCAATAG |
| CER13_Seq_S20 | AAGAGTCCAAGTCATTTCTCAGAAG |
| CER13_Seq_R1 | TATAGCCTGCAGATTAAAGAAGTTC |
| CER13_Seq_R2 | AGAATACAATAGATGGGATTTGCA |
| CER13_Seq_R15 | AAAAGTTAGGAGGCTTCAGTGC |
| CER13_Seq_R16 | ACGTAATTAACCTAAACCCTAACAT |
| CER13_Seq_R17 | TGAAGAGGGTTTCCTGGAATAT |
| CER13_Seq_R18 | TACAGATGACAAGCTTCTCATCG |
|  |  |
| CER8_Seq_S1 | CCTTCCCCATACAATTTATATCTAG |
| CER8_Seq_S2 | TGATTTGAGGTTTTAGGTATTTGAA |
| CER8_Seq_S3 | AACTATTTCATAGCAGAACTAACGC |
| CER8_Seq_S4 | TAAAAGGCTTGAACACTGTATCATC |
| CER8_Seq_S5 | CTCAATCTGCACAATAAGGAATTAC |
| CER8_Seq_S6 | TTCCCAATCTTCAGATGTATTATTG |
| CER8_Seq_S7 | GCCTTTATGCTAAATATGGTATCC |
| CER8_Seq_R7 | AGGATACCATATTTAGCATAAAGGC |
| CER8_Seq_S8 | GCAGATACTTGGCTATAAACCTTAT |
| CER8_Seq_S9 | TTATAAATTCAGTAGTCTGAGGCAT |
| CER8_Seq_S10 | GAAACTCTTTATGACTCTGAAGGAA |
| CER8_Seq_S11 | AATAACTCACCTGAAGAATCTATGC |
| CER8_Seq_R11 | TTACTGATACCAAGATGTCGACTCT |
| CER8_Seq_S12 | TGGATAGAAGATAAGAAAGTGGATG |
| CER8_Seq_S13 | GGAAAGTTGTCAGAAGAAAATCTTT |
| CER8_Seq_R13 | TTAATGTTAAGGCTGACTATATCCA |
| CER8_Seq_S14 | AGATTTGCTCTCTGGACTGAATAAT |
| CER8_Seq_R14 | GGGATAATAGGAATCAGATATGCTC |
| CER8_Seq_S15 | AAAGAGCATGGGTTCTAGTGAAA |
| CER8_Seq_S16 | GCAGATACCCTTATTTACCAGATAA |
| CER8_Seq_S17 | TTTCAGTCCCTCTATACATCATGTG |
| CER8_Seq_S18 | GAATGGGTTATCTGTCTTCTCAAG |
| CER8_Seq_S19 | AATATTAAGGAAGTTATGGAATGGC |
| CER8_Seq_S20 | CCCATATAATCTCATGGTGGTTATA |
| CER8_Seq_S21 | TGTCCACTCTTTTCACATTTTTCT |
| CER8_Seq_S22 | TTTTGATACCCTAACTTGAGCTACA |
|  |  |
| CER10_Seq_S1 | CAACATATCCCTCAATGAAAATCTA |
| CER10_Seq_S2 | CACAAGAACTGAAGATCTATCCTGA |
| CER10_Seq_S3 | TGTGGTATTGCAATTAAAACAATTA |
| CER10_Seq_S4 | CAGTGACCTGAAATTATTGACTGAC |
| CER10_Seq_S5 | TCTAGAGACATGGTCTCATTCTGTT |
| CER10_Seq_S6 | CACTTTCTTATGTTATTAAATAGCC |
| CER10_Seq_S7 | GCCTCATGTTTTCTTTAAGCAGT |
| CER10_Seq_R7 | GTTTATGTTGGTCAAAAATAGGAAA |
| CER10_Seq_S8 | GAACATAGTTTCCTATTTTTGACCA |
| CER10_Seq_S9 | ATAGAAGGGGCTAGGAAATGAAG |
| CER10_Seq_R9 | CTTCATTTCCTAGCCCCTTCTAT |
| CER10_Seq_S10 | ATGCTCAGACTGAAGAAAGTAATTG |
| CER10_Seq_S11 | GAAGGACCTGTTCAAAGTATTTTCT |
| CER10_Seq_S12 | AGGTCACAAAGATAAGCAGAGAGA |
| CER10_Seq_S13 | TCAGAATCTAAAGGGAGTTTATTCA |
| CER10_Seq_S14 | GTGTGTTTAACCTTTCACACTGAAT |
| CER10_Seq_S15 | CTATACTACTTTGTTGAGCACAGCA |
| CER10_Seq_R15 | CAACAGGACAATTCTGGATAAAAC |
| CER10_Seq_S16 | TCACTTCCTTCAAACTATCGATAAG |
| CER10_Seq_R16 | AGTTGAAAAGAACAGCATCAGTTAG |
| CER10_Seq_S17 | AATCTTGATCTCTTGAACTTGTGAT |
| CER10_Seq_R17 | TTCAAGAGATCAAGATTATACTGGC |
| CER10_Seq_S18 | AAACTACTCCAAACCTAGTGACTTA |
| CER10_Seq_S19 | TACTTAGGATCTCCATTTCGTGG |
| CER10_Seq_S20 | ATTCCATGACTCAATGGATAACAT |
| CER10_Seq_S21 | GGCTGCATACATTTCATCATATG |
|  |  |
| CER6_Seq_S1 | GCAGGACCTGGATTCTTCTAATAT |
| CER6_Seq_S2 | AAATCTAGAAAGATTCCGGAGG |
| CER6_Seq_S3 | GACATAAAATCTGGAGATTGTATGC |
| CER6_Seq_S4 | AGCCAGACTAAAAGTGTTGTAGTCA |
| CER6_Seq_S5 | GAATTTGGAAGAAGTACTTAGGGTT |
| CER6_Seq_S6 | TCAAGGTACATCATCTGGTTAAATC |
| CER6_Seq_S7 | AGAATGTGTTCTGCAAGTTTAATTT |
| CER6_Seq_S8 | GAACAAATAAATATGACCTCAATGG |
| CER6_Seq_S9 | TATCATCTCCACAACTGTCTAATCC |
| CER6_Seq_S10 | ATCCACCTGTCAACTAGGTTTTAA |
| CER6_Seq_S11 | TGATTGTAGATCCTTGAGGAATTG |
| CER6_Seq_S12 | TGCCTATTCACTCTGATGATAGCT |
| CER6_Seq_S13 | TATACATCTCCTCTTGTCATTACCC |
| CER6_Seq_S14 | TGTGGAACTGTAAATGATACACACA |
| CER6_Seq_R14 | CAGTATAAGGTCAAACAATTCACTG |
| CER6_Seq_S15 | AGCAGTACTTCAAACTTTGACAATT |
| CER6_Seq_R15 | ACCAGGCTACAAAAAAGTCTTCA |
| CER6_Seq_S16 | GTTTGTTTTTATGTTTCCAAACTCA |
| CER6_Seq_R16 | GCCTTTTACTCTGATCATTCTCTTT |
| CER6_Seq_S17 | GTATAATTGCCAATCACGTGGT |
| CER6_Seq_R17 | CATTTAACAAGCAGTTTAATCCACT |
| CER6_Seq_S18 | TTGATATTTCAGTTTTGTTAGCAAA |
| CER6_Seq_R18 | AATTGCGAACATGTAGTTGTGAG |
| CER6_Seq_S19 | CAAAGAATGGAACATTTTCAGTGT |
| CER6_Seq_R19 | CCACAGTCACTGATCTACATTGAAT |
| CER6_Seq_S20 | GCATTGAAACTCTCTCAACCTTC |
| CER6_Seq_S21 | GTCTTGTCAGAAACTGAAACAACTG |
|  |  |
| CER3_Seq_S1 | CAGAATACATGCATCTTGGATGTA |
| CER3_Seq_S2 | CTCTAGAAGCTTTTACTTATGGCAA |
| CER3_Seq_S3 | TTACTGAGTTCAGGAAGTTGATCTG |
| CER3_Seq_S4 | GAGAATCACTTGAACCTGGAAGA |
| CER3_Seq_S5 | TCTATATTTGCTTGGACAATTGACT |
| CER3_Seq_S6 | AGAGAATTATACCTCCCTGAAACAT |
| CER3_Seq_S7 | GTGCATTGTTGTATTACTCAGGATT |
| CER3_Seq_S8 | ATTTTATTATATTCAGGCCCTCAAT |
| CER3_Seq_S9 | ACAAACAGAATGGGATATAAATGG |
| CER3_Seq_S10 | ACAAGAAATTCTTTATAGCTGTCCA |
| CER3_Seq_S11 | ACTGGAGATGGTTCAGAAAAGAAT |
| CER3_Seq_S12 | TACAGCTCCTTTATTCTAACGGG |
| CER3_Seq_S13 | AAACCTCCCTAGTATCTTAAGTTGG |
| CER3_Seq_S14 | GCACCTATCATAGAAAGATGATCAT |
| CER3_Seq_S15 | TGGCCATCTTCATAATATTTTTATG |
| CER3_Seq_S16 | AGTATGTTTGAAGGTGATAAGTGCTT |
| CER3_Seq_S17 | CAGTTGAACCAAGGATGAATGAT |
| CER3_Seq_S18 | CTATTGTATTATGGTTCAGGGAAGA |
| CER3_Seq_S19 | ACGTGAATAAATGACACAGTACAGC |
| CER3_Seq_S20 | AGTCAAGAATATTTTGGTGTGTCAT |
| CER3_Seq_S21 | GTGTGGTAACACAGGCAAATATAA |
| CER3_Seq_S22 | AAGAAAGGAAGCTGTATCATACCA |
| CER3_Seq_S23 | TTGTCGTCTGAATATTTGTATCTCC |
| CER3_Seq_S24 | CAATAGGACTGAGACACACCTATGT |
| CER3_Seq_S25 | ACAATGAGTGGCAGTCTCTTTTC |
| CER3_Seq_S26 | GATTTACACTAGGACCTTTGTGAAA |
| CER3_Seq_S27 | TGTACTCAGACACTTTAGCTTACGG |

Supplementary Table 4 Reconstructed phylogenetic trees in newick format

CER3:

((((((((((((CE14_1:0.00589667,CE14_2:0.00460941):0.02428280,(RMC16_1:0.00870456,RMC16_2_null:0.01164321):0.01329525):0.00730973,((RMC08_1_null:0.01047451,RMC08_2_null:0.00546911):0.02389059,(RMC14_1:0.00985520,RMC14_2:0.00624741):0.01408888):0.00359384):0.00044667,((CE08_1:0.00780318,CE08_2_null:0.00839602):0.00865989,((CE17_1_null:0.00711640,CE17_2:0.01486990):0.01532471,(RMC17_1_null:0.01176802,RMC17_2_null:0.01112892):0.02143894):0.00513182):0.00593572):0.00205053,(((CE12_1:0.00562508,CE12_2:0.00393163):0.03473230,(RMC12_1:0.02498485,RMC12_2:0.00489552):0.01607369):0.01078430,((CE09_1:0.01339831,CE09_2:0.00741054):0.02058250,((CE16_1_null:0.01623457,CE16_2_null:0.01397683):0.02351750,(RMC09_1_null:0.01641057,RMC09_2:0.00927906):0.01628602):0.00918938):0.00326697):0.00413022):0.00409218,(((CE05_1:0.00517652,CE05_2_null:0.00735980):0.01890521,(RMC13_1:0.01765524,RMC13_2:0.00741289):0.02303671):0.00546363,((CE13_1_null:0.01095988,CE13_2_null:0.01444827):0.02118906,(((CE15_1:0.00719203,CE15_2:0.01848627):0.01679912,(RMC15_1:0.02136041,RMC15_2_null:0.01536069):0.01906751):0.00725676,(((CE11_1_null:0.00853838,CE11_2_null:0.00948167):0.01470667,(RMC05_1:0.00553173,RMC05_2_null:0.00828259):0.02913431):0.00454773,((RMC11_1:0.00635076,RMC11_2:0.00958653):0.01635938,((CE18_1:0.00941920,CE18_2:0.01002830):0.02453129,(RMC18_1_null:0.01133842,RMC18_2:0.01153239):0.01212983):0.00733995):0.00345890):0.00306154):0.00128391):0.00307763):0.00000000):0.00258480,((CE19_1:0.00949009,CE19_2:0.01007507):0.01882919,(RMC19_1:0.00514729,RMC19_2:0.01100531):0.01355867):0.00388644):0.00519395,((CE02_1:0.00594094,CE02_2:0.01549833):0.01710237,((((CE10_1_null:0.01115254,CE10_2_null:0.00601263):0.01408917,(RMC10_1_null:0.01157076,RMC10_2:0.01129687):0.01936898):0.01307779,(CE04_1:0.01620943,CE04_2:0.01276276):0.01161870):0.00289137,((RMC04_1:0.01863125,RMC04_2:0.00301041):0.03881271,((CE07_1:0.01352038,CE07_2:0.00753020):0.02681081,(RMC07_1_null:0.01396408,RMC07_2_null:0.00000006):0.02951263):0.01079649):0.00787505):0.00125512):0.00605245):0.00130382,(((CE06_1_null:0.02424785,CE06_2:0.01185666):0.01312786,(RMC06_1_null:0.01612798,RMC06_2_null:0.00803211):0.02008317):0.00774843,(((CE03_1:0.01123545,CE03_2:0.01962270):0.01657723,(RMC03_1_null:0.02004482,RMC03_2_null:0.01069967):0.01712848):0.00953958,((CE20_1:0.01556480,CE20_2:0.01575445):0.03521117,(RMC20_1_null:0.01744685,RMC20_2_null:0.00849828):0.01362970):0.00516519):0.00565482):0.00724628):0.00097883,(RMC02_1_null:0.01261121,RMC02_2_null:0.01902632):0.01823694):0.00000000,((CE01_1:0.01376294,CE01_2:0.01145803):0.01054656,(RMC01_1_null:0.00846635,RMC01_2_null:0.00750672):0.00404083):0.00268126):1.33980288,Retro21_bb:1.41471212);

CER6:

((((((((((((((((((((((CE02_1_null:0.00847846,CE02_2:0.01946621):0.01511964,(CE18_1:0.00518681,CE18_2_null:0.01018246):0.03324516):0.01098303,(CE19_1:0.00807998,CE19_2:0.00596988):0.01267701):0.00811498,((CE04_1:0.01465648,CE04_2:0.01477879):0.02304978,(CE17_1:0.00553430,CE17_2_null:0.01313232):0.01348624):0.01302451):0.00351235,((CE20_1:0.00419705,CE20_2_null:0.01452181):0.01794374,((CE12_1:0.01219953,CE12_2:0.01094310):0.00835717,(CE14_1:0.00666324,CE14_2:0.00852137):0.02154063):0.00509632):0.00454818):0.00928002,(CE16_1_null:0.01545514,CE16_2:0.03465814):0.01474925):0.01121836,(((CE06_1:0.04288316,CE06_2:0.01096491):0.01597195,(CE09_1_null:0.03083112,CE09_2:0.02555473):0.02904199):0.01195998,((CE08_1:0.01632585,CE08_2:0.01170597):0.02286622,((CE07_1:0.01515672,CE07_2_null:0.00708989):0.02406039,(CE10_1:0.01470145,CE10_2:0.01449289):0.04899700):0.01571947):0.01252361):0.01165852):0.02077834,(((RMC03_1:0.01728097,RMC03_2:0.01777387):0.01692992,(RMC13_1:0.00780847,RMC13_2:0.01053124):0.02005650):0.01094086,((RMC14_1:0.01060749,RMC14_2:0.00452171):0.01472295,((RMC02_1:0.01288350,RMC02_2:0.02791871):0.01707672,(RMC12_1_null:0.00956542,RMC12_2:0.02664817):0.01819290):0.02331571):0.01103087):0.00734677):0.00319086,(RMC04_1_null:0.00861584,RMC04_2:0.01745062):0.02453097):0.02024343,((RMC06_1:0.01191190,RMC06_2_null:0.02643859):0.01982320,(RMC16_1_null:0.00647071,RMC16_2:0.00864360):0.02406501):0.00814717):0.01093295,(((RMC01_1:0.01437255,RMC01_2:0.02025412):0.01981182,(RMC11_1_null:0.02179299,RMC11_2:0.00846296):0.03432864):0.00504326,((RMC05_1:0.02556184,RMC05_2:0.03007139):0.02784685,(RMC15_1:0.01366046,RMC15_2:0.01173001):0.01255105):0.00889783):0.00223104):0.00588319,((RMC07_1:0.02686610,RMC07_2:0.03180332):0.03823484,(RMC17_1:0.00457364,RMC17_2:0.02645165):0.01604928):0.01347242):0.01977612,(RMC20_1:0.01631349,RMC20_2:0.00000000):0.00999864):0.00656481,((RMC10_1:0.01831146,RMC10_2:0.01255800):0.02814509,((RMC19_1_null:0.00519364,RMC19_2:0.00547514):0.00892626,((RMC09_1:0.00615777,RMC09_2:0.01836542):0.03877804,((RMC08_1:0.01569122,RMC08_2_null:0.01122854):0.04178128,(RMC18_1:0.00098593,RMC18_2:0.00875944):0.01464573):0.01415077):0.01071650):0.00628860):0.00974465):0.02450820,((CE01_1_null:0.01264169,CE01_2:0.02981690):0.03153013,(CE05_1:0.00894956,CE05_2:0.01764985):0.01091162):0.01143314):0.00655001,CE15_2:0.02117914):0.03130908,CE15_1:0.00999606):0.03812664,(CE11_1:0.01017397,CE11_2:0.01201275):0.02977732):0.01739061,(CE13_1_null:0.00594923,CE13_2:0.00248439):0.01589887):0.00705807,CE03_1:0.01340330):0.00304385,CE03_2:0.00909378):1.45675850,Retro20_bb:1.77571469);

CER8:

((((((((((((((((CE07_1_null:0.01224758,CE07_2_null:0.01069288):0.01658812,CE02_1_null:0.01119344):0.00403838,(CE06_1_null:0.00576413,CE06_2_null:0.00828078):0.01616537):0.00481854,(CE08_1:0.00961204,CE08_2:0.00956662):0.01393240):0.00393022,CE02_2_null:0.00659117):0.00689071,((CE11_1_null:0.01093625,CE11_2_null:0.00608374):0.02683670,(RMC19_1:0.00791573,RMC19_2_null:0.01240492):0.01002820):0.00278430):0.00209548,((CE14_1_null:0.00933201,CE14_2_null:0.00677735):0.01022540,(CE20_1:0.02962094,CE20_2:0.02618367):0.01637962):0.01375039):0.00225681,(((CE05_1_null:0.01422334,CE05_2_null:0.00918191):0.01709895,(CE15_1_null:0.00874347,CE15_2_null:0.02353224):0.01677291):0.00565652,((CE17_1:0.01402239,CE17_2_null:0.00887117):0.01506289,((CE03_1_null:0.00904128,CE03_2_null:0.00742421):0.01412758,(CE13_1_null:0.00000243,CE13_2_null:0.01635288):0.03125849):0.01008495):0.00346714):0.00484157):0.00459341,(CE16_1:0.01590463,CE16_2_null:0.00447983):0.01939272):0.01176867,((CE01_1_null:0.01683159,CE01_2_null:0.01775314):0.02320508,((RMC05_1_null:0.02187957,RMC05_2_null:0.00309669):0.02479874,((RMC03_1:0.00866642,RMC03_2:0.00666663):0.01310288,(RMC17_1_null:0.00306884,RMC17_2_null:0.00832229):0.00441019):0.00441771):0.00000000):0.00224430):0.00200353,((((CE10_1_null:0.00673448,CE10_2_null:0.01798953):0.01080549,(CE19_1_null:0.01417334,CE19_2_null:0.00863542):0.01712532):0.01062798,(CE18_1_null:0.01271613,CE18_2_null:0.01178601):0.01667230):0.00879840,((RMC08_1_null:0.00674744,RMC08_2_null:0.01163263):0.01401503,((RMC06_1:0.00179049,RMC06_2:0.00822060):0.00877568,(RMC07_1_null:0.00132938,RMC07_2_null:0.01235678):0.01763448):0.00816482):0.00688136):0.00444600):0.00528741,(((CE04_1_null:0.00108437,CE04_2_null:0.01970568):0.01626392,(RMC02_1_null:0.01563216,RMC02_2_null:0.01234423):0.01559060):0.00801094,(RMC01_2_null:0.00431904,(RMC01_1_null:0.01124009,((RMC04_1_null:0.01917580,RMC04_2_null:0.02001582):0.01252161,((RMC09_1_null:0.04266673,RMC09_2_null:0.01175648):0.02825424,(RMC12_1_null:0.04338938,RMC12_2_null:0.01774227):0.03159880):0.02437423):0.02424800):0.01249121):0.01729481):0.00306098):0.00334733,((((CE09_1_null:0.01542702,CE09_2_null:0.01265003):0.02628879,(RMC15_1_null:0.01750624,RMC15_2_null:0.02013487):0.00925700):0.01181581,((CE12_1:0.01044179,CE12_2:0.02644863):0.01741710,(RMC14_1_null:0.01595115,RMC14_2_null:0.00651567):0.02386809):0.00759528):0.02086978,((((RMC13_1_null:0.02555901,RMC13_2:0.02392728):0.03156907,RMC16_2:0.00777460):0.01426169,RMC16_1_null:0.00746560):0.01066898,((RMC20_1:0.01194285,RMC20_2:0.00884479):0.02290736,((RMC10_1:0.00853405,RMC10_2:0.02290646):0.01750393,(RMC11_1_null:0.01214558,RMC11_2_null:0.02979689):0.01486375):0.01474543):0.00801540):0.00661809):0.00460409):0.02030533,RMC18_1_null:0.00433468):0.00000010,RMC18_2_null:0.02158236):1.09861477,Retro7_bb:1.27872495);

CER10:

((((((((((((((((((((((((((((RMC04_2_null:0.00000000,RMC07_1_null:0.00113777):0.00033567,RMC04_1_null:0.00080197):0.00044645,(RMC14_1_null:0.00075362,RMC14_2_null:0.00054776):0.00062928):0.00071377,RMC13_1_null:0.00049907):0.00031315,RMC13_2_null:0.00016211):0.00034173,((RMC09_1_null:0.00073830,RMC09_2_null:0.00072704):0.00065842,(RMC17_1_null:0.00105321,RMC17_2_null:0.00041182):0.00056324):0.00055380):0.00048478,(RMC16_1_null:0.00047437,((RMC03_1_null:0.00117642,RMC03_2_null:0.00094393):0.00035832,((RMC02_1_null:0.00029565,RMC02_2_null:0.00068018):0.00095168,(RMC12_1_null:0.00095206,RMC12_2_null:0.00018589):0.00019008):0.00037915):0.00089039):0.00000000):0.00047088,(RMC19_1_null:0.00127406,RMC19_2_null:0.00035457):0.00048824):0.00051230,(RMC11_2_null:0.00049486,(RMC20_1_null:0.00049402,(RMC11_1_null:0.00085854,(RMC01_1_null:0.00024173,RMC01_2_null:0.00105962):0.00085189):0.00038573):0.00034901):0.00063336):0.00066394,(RMC10_2_null:0.00016228,(RMC10_1_null:0.00020243,(RMC15_1_null:0.00060825,RMC15_2_null:0.00052753):0.00065221):0.00044671):0.00032480):0.00026771,RMC20_2_null:0.00054569):0.00048617,(RMC07_2_null:0.00090563,(RMC08_2_null:0.00056628,(RMC08_1_null:0.00000000,(RMC16_2_null:0.00048819,(RMC18_1_null:0.00000000,RMC18_2_null:0.00064942):0.00081407):0.00032469):0.00057456):0.00032216):0.00083806):0.00027179,(RMC05_1_null:0.00019863,(RMC05_2_null:0.00052342,RMC06_1_null:0.00077962):0.00032504):0.00056572):0.00048982,RMC06_2_null:0.00086746):0.00108838,CE18_2_null:0.00000000):0.00048729,CE18_1_null:0.00016242):0.00047407,(CE19_1_null:0.00018081,CE19_2_null:0.00095794):0.00167257):0.00063000,(CE09_1_null:0.00000000,CE09_2_null:0.00129987):0.00035828):0.00078347,(((CE17_1_null:0.00034900,CE17_2_null:0.00079063):0.00065529,CE20_2_null:0.00111525):0.00051262,(((CE08_1_null:0.00049184,CE08_2_null:0.00032004):0.00016058,CE11_1_null:0.00114383):0.00016284,((CE06_1_null:0.00071665,CE06_2:0.00107600):0.00096774,((CE10_1_null:0.00110851,CE10_2_null:0.00019529):0.00110501,((CE11_2_null:0.00084118,CE14_2_null:0.00029684):0.00029636,((CE12_1_null:0.00032517,CE12_2_null:0.00048757):0.00032428,(CE14_1_null:0.00048747,(CE13_1_null:0.00000000,CE13_2_null:0.00064953):0.00032481):0.00016173):0.00021818):0.00018237):0.00038287):0.00042646):0.00049018):0.00016096):0.00048851,((CE15_1_null:0.00016472,CE15_2_null:0.00064551):0.00048695,(CE20_1_null:0.00066090,(CE05_1:0.00067301,CE05_2:0.00046357):0.00048762):0.00064234):0.00000000):0.00048340,(CE16_1_null:0.00048815,CE16_2_null:0.00065120):0.00064908):0.00000000,(CE07_1:0.00130469,CE07_2_null:0.00016111):0.00032531):0.00097559,CE03_1:0.00000000):0.00054667,CE03_2:0.00043012):0.00097914,(CE04_1:0.00000000,CE04_2:0.00113953):0.00075686):0.00038538,(CE02_2:0.00058161,(CE01_1:0.00055742,CE01_2:0.00074290):0.00032562):0.00055883):0.00048719,CE02_1:0.00000000):0.00380872,Retro9_bb:0.01855374);

CER11:

((((((((((((((((((((((CE11_1_null:0.00177102,CE11_2_null:0.00066095):0.00090942,(CE16_1:0.00176804,CE16_2:0.00111113):0.00108318):0.00044668,CE17_2_null:0.00067328):0.00067483,(CE17_1_null:0.00111902,(CE7_1:0.00086287,CE7_2:0.00224315):0.00117840):0.00126455):0.00104353,CE18_2:0.00108474):0.00108256,(CE9_1_null:0.00164693,CE9_2_null:0.00123000):0.00103388):0.00130372,CE18_1:0.00186747):0.00101222,(CE19_1_null:0.00173547,CE20_1_null:0.00224493):0.00083641):0.00116122,((CE6_1:0.00220649,CE6_2:0.00065841):0.00046869,(RMC6_1_null:0.00220974,RMC6_2_null:0.00065959):0.00219156):0.00066172):0.00097498,(CE5_2_null:0.00045813,(CE5_1_null:0.00097542,(CE14_1_null:0.00120143,CE14_2_null:0.00099801):0.00112697):0.00120354):0.00090876):0.00057198,(CE4_1_null:0.00115872,CE4_2_null:0.00103774):0.00107611):0.00182004,CE20_2_null:0.00134233):0.00090954,((CE10_1_null:0.00040900,CE10_2_null:0.00113394):0.00169630,(CE19_2_null:0.00231080,(CE8_1_null:0.00025994,CE8_2:0.00150529):0.00140850):0.00067442):0.00153997):0.00037740,(((RMC10_1_null:0.00126924,RMC10_2_null:0.00159692):0.00087817,(RMC12_1_null:0.00171713,RMC12_2_null:0.00048459):0.00122565):0.00036474,((((RMC3_1_null:0.00192034,RMC3_2_null:0.00117326):0.00198774,RMC2_1_null:0.00191764):0.00111566,RMC2_2_null:0.00095097):0.00090789,(RMC5_1_null:0.00065492,(RMC5_2_null:0.00132958,((RMC4_1_null:0.00121152,RMC4_2_null:0.00055078):0.00183571,(RMC11_1_null:0.00069329,RMC11_2_null:0.00194850):0.00188264):0.00138276):0.00000000):0.00119243):0.00072497):0.00088806):0.00123382,((RMC9_1_null:0.00111991,RMC9_2_null:0.00130425):0.00170073,(((RMC7_1_null:0.00109626,RMC7_2_null:0.00155506):0.00217922,RMC13_2_null:0.00158369):0.00084597,(RMC13_1_null:0.00113832,(RMC14_1_null:0.00090484,RMC14_2_null:0.00107247):0.00177204):0.00025215):0.00066781):0.00025418):0.00066346,((CE2_1_null:0.00156245,CE2_2_null:0.00063960):0.00110214,(CE3_1_null:0.00155737,CE3_2_null:0.00065159):0.00112275):0.00156713):0.00000000,(RMC17_1_null:0.00084269,RMC17_2_null:0.00136003):0.00131886):0.00057088,((RMC15_1_null:0.00242372,RMC15_2_null:0.00023102):0.00093001,(RMC16_1_null:0.00154996,RMC16_2_null:0.00109307):0.00170470):0.00045260):0.00000000,(RMC8_1_null:0.00152702,(RMC8_2_null:0.00121256,(CE13_1_null:0.00143968,(CE13_2_null:0.00115119,(CE15_1_null:0.00151545,CE15_2_null:0.00024616):0.00168959):0.00076224):0.00117584):0.00073160):0.00116166):0.00076353,(RMC18_1_null:0.00065797,RMC18_2_null:0.00110213):0.00104495):0.00042469,((RMC20_1_null:0.00000000,RMC20_2_null:0.00065869):0.00207436,((RMC1_1_null:0.00070823,RMC1_2_null:0.00284830):0.00204520,((CE12_1_null:0.00140303,CE12_2_null:0.00080450):0.00093212,((CE1_1_null:0.00060295,CE1_2_null:0.00049594):0.00096658,(RMC19_1_null:0.00030028,RMC19_2_null:0.00102043):0.00071672):0.00077604):0.00033671):0.00043832):0.00088667):0.00121353,Retro3_bb:0.01981321);

CER13:

((((((((((CE15_1_null:0.00070510,CE15_2_null:0.00070461):0.00013842,CE02_1_null:0.00042176):0.00056309,(CE02_2:0.00112756,CE03_1:0.00028005):0.00056352):0.00000000,CE03_2:0.00028072):0.00023707,((CE04_1:0.00095404,CE04_2:0.00031432):0.00035727,((CE11_1:0.00028106,CE11_2:0.00028065):0.00081038,(CE12_1_null:0.00079069,CE12_2:0.00047803):0.00037228):0.00063256):0.00053150):0.00029729,CE19_2:0.00137055):0.00017113,(CE19_1:0.00111494,(((CE18_1:0.00032322,CE18_2:0.00080117):0.00097429,CE01_1:0.00075970):0.00080589,(CE01_2_null:0.00068757,(CE13_1_null:0.00053641,CE13_2_null:0.00016645):0.00097769):0.00059031):0.00000000):0.00030078):0.00042742,(((((CE05_1_null:0.00056051,CE05_2_null:0.00056470):0.00035831,(CE06_1:0.00042133,CE06_2:0.00028129):0.00105342):0.00052817,(CE17_1:0.00094725,CE17_2:0.00017937):0.00115668):0.00057272,(CE10_2:0.00053316,(CE10_1_null:0.00045608,(CE14_1_null:0.00042068,CE14_2_null:0.00042264):0.00081178):0.00014085):0.00031538):0.00029495,(((CE07_1:0.00033387,CE07_2_null:0.00065175):0.00109184,(CE08_1:0.00022775,CE08_2:0.00019351):0.00045945):0.00029373,((CE16_1:0.00017527,CE16_2_null:0.00095103):0.00057000,(CE09_2_null:0.00042229,(CE09_1:0.00000000,(CE20_1:0.00127005,CE20_2:0.00042034):0.00098792):0.00042150):0.00080286):0.00039700):0.00033066):0.00026478):0.00043814,(((((((((RMC05_1_null:0.00054274,RMC05_2:0.00015980):0.00084561,RMC03_2_null:0.00026182):0.00015969,RMC03_1:0.00042154):0.00028123,(RMC13_1:0.00000001,RMC13_2_null:0.00028083):0.00028145):0.00042179,(RMC08_2_null:0.00112637,RMC18_2:0.00000000):0.00042243):0.00046064,((RMC01_1:0.00000000,RMC01_2:0.00014022):0.00031936,(RMC11_1:0.00038262,RMC11_2:0.00017872):0.00014043):0.00034390):0.00033237,(RMC15_1_null:0.00042122,RMC15_2_null:0.00000000):0.00075522):0.00017950,(RMC19_1:0.00016018,(RMC19_2_null:0.00019892,(RMC09_1_null:0.00066695,RMC09_2_null:0.00045789):0.00026060):0.00038359):0.00063623):0.00087709,(((RMC10_1_null:0.00041015,RMC10_2_null:0.00114091):0.00065514,(RMC20_1:0.00088884,RMC20_2:0.00066074):0.00055473):0.00025629,(((RMC02_1_null:0.00056280,RMC02_2_null:0.00028102):0.00031421,RMC12_2:0.00081191):0.00078671,((RMC08_1_null:0.00046082,RMC18_1:0.00123088):0.00067440,(((RMC07_1:0.00014398,RMC07_2_null:0.00055814):0.00056321,(RMC17_1_null:0.00028159,RMC17_2_null:0.00028027):0.00014365):0.00033337,((RMC16_1_null:0.00045363,RMC16_2_null:0.00067310):0.00034586,((RMC04_1_null:0.00042155,RMC04_2_null:0.00028123):0.00051249,((RMC14_1:0.00016324,RMC14_2:0.00039845):0.00024119,(RMC12_1:0.00000000,(RMC06_1_null:0.00028057,RMC06_2_null:0.00070260):0.00084566):0.00062544):0.00063148):0.00037328):0.00046892):0.00037328):0.00050522):0.00048248):0.00048160):0.00014041):0.00601038,Retro4_bb:0.01164076);

Supplementary Figure 1 Phylogenetic tree of CER3


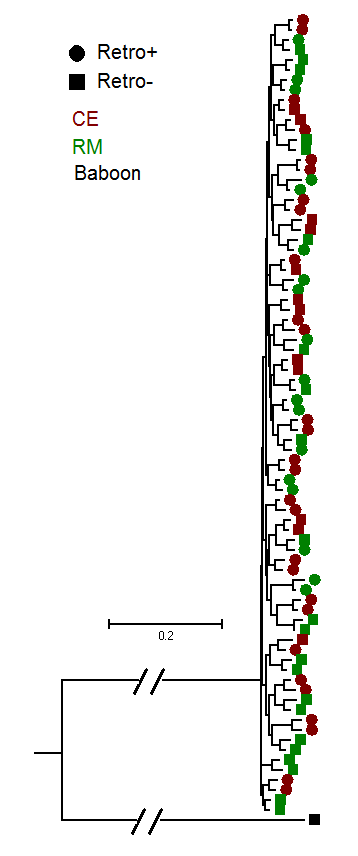


Supplementary Figure 2 Phylogenetic tree of CER8


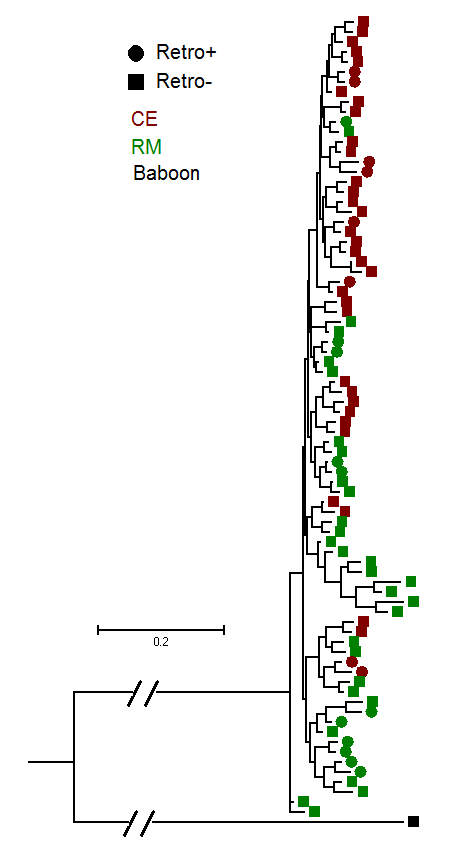


Supplementary Figure 3 Phylogenetic tree of CER10


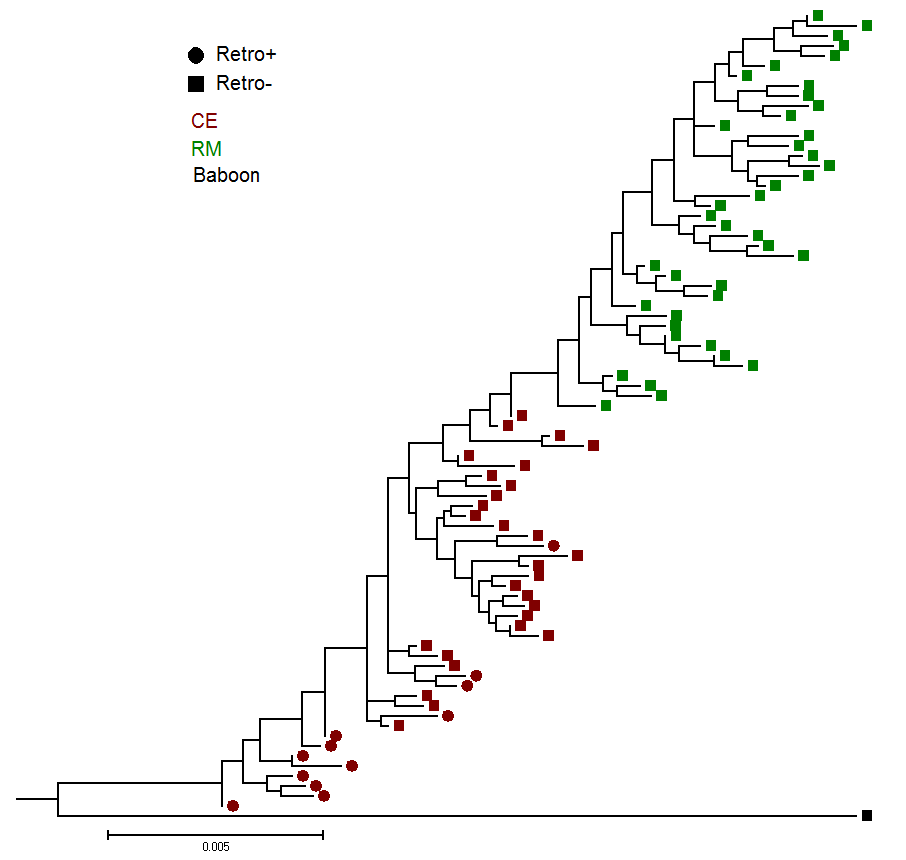


Supplementary Figure 4 Phylogenetic tree of CER11


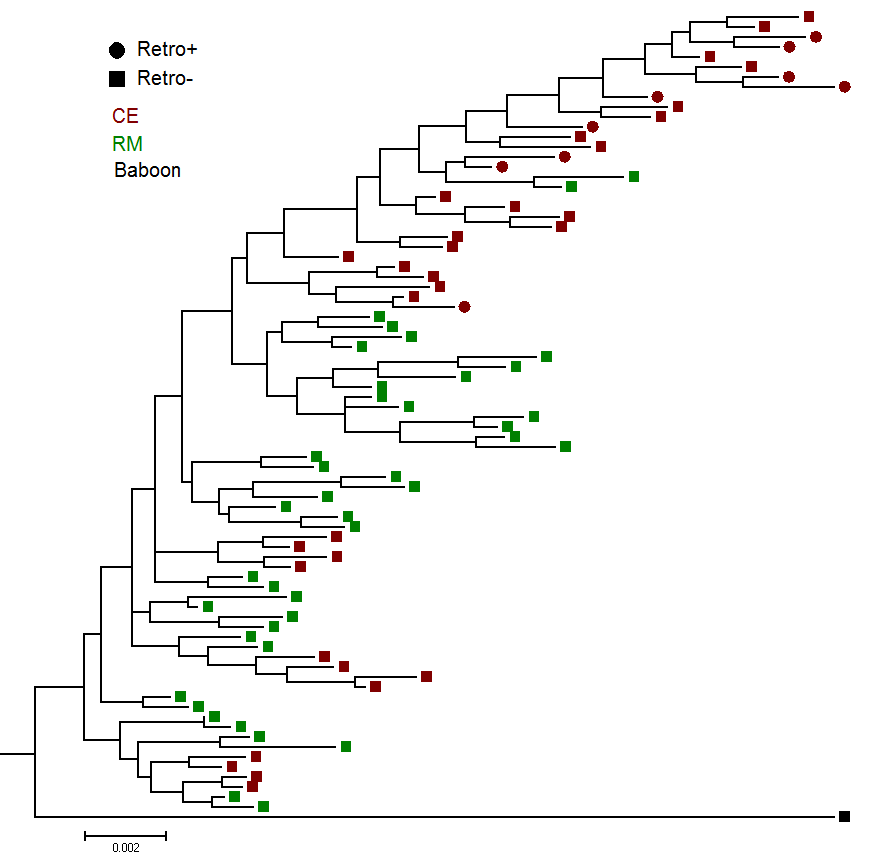

Supplement: Supplementary Information [file srep32598-s1.doc]
